# The Effects of Crude Oil and Dispersant on the Larval Sponge Holobiont

**DOI:** 10.1128/mSystems.00743-19

**Published:** 2019-12-10

**Authors:** Heidi M. Luter, Steve Whalan, Nikos Andreakis, Muhammad Abdul Wahab, Emmanuelle S. Botté, Andrew P. Negri, Nicole S. Webster

**Affiliations:** a Australian Institute of Marine Science, Townsville, Queensland, Australia; b Marine Ecology Research Centre, School of Environment, Science and Engineering, Southern Cross University, Lismore, New South Wales, Australia; c College of Science and Engineering, James Cook University, Townsville, Queensland, Australia; d Australian Centre for Ecogenomics, University of Queensland, Brisbane, Queensland, Australia; University of California, Riverside

**Keywords:** sponge larvae, hydrocarbon toxicity, gene expression, microbial symbiosis

## Abstract

Accidental oil spills from shipping and during extraction can threaten marine biota, particularly coral reef species which are already under pressure from anthropogenic disturbances. Marine sponges are an important structural and functional component of coral reef ecosystems; however, despite their ecological importance, little is known about how sponges and their microbial symbionts respond to petroleum products. Here, we use a systems biology-based approach to assess the effects of water-accommodated fractions (WAF) of crude oil, chemically enhanced water-accommodated fractions of crude oil (CWAF), and dispersant (Corexit EC9500A) on the survival, metamorphosis, gene expression, and microbial symbiosis of the abundant reef sponge Rhopaloeides odorabile in larval laboratory-based assays. Larval survival was unaffected by the 100% WAF treatment (107 μg liter^−1^ polycyclic aromatic hydrocarbon [PAH]), whereas significant decreases in metamorphosis were observed at 13% WAF (13.9 μg liter^−1^ PAH). The CWAF and dispersant treatments were more toxic, with decreases in metamorphosis identified at 0.8% (0.58 μg liter^−1^ PAH) and 1.6% (38 mg liter^−1^ Corexit EC9500A), respectively. In addition to the negative impact on larval settlement, significant changes in host gene expression and disruptions to the microbiome were evident, with microbial shifts detected at the lowest treatment level (1.6% WAF; 1.7 μg liter^−1^ PAH), including a significant reduction in the relative abundance of a previously described thaumarchaeal symbiont. The responsiveness of the R. odorabile microbial community to the lowest level of hydrocarbon treatment highlights the utility of the sponge microbiome as a sensitive marker for exposure to crude oils and dispersants.

**IMPORTANCE** Larvae of the sponge R. odorabile survived exposure to high concentrations of petroleum hydrocarbons; however, their ability to settle and metamorphose was adversely affected at environmentally relevant concentrations, and these effects were paralleled by marked changes in sponge gene expression and preceded by disruption of the symbiotic microbiome. Given the ecological importance of sponges, uncontrolled hydrocarbon releases from shipping accidents or production could affect sponge recruitment, which would have concomitant consequences for reef ecosystem function.

## Abstract (precis)

Larvae of the sponge R. odorabile survived exposure to high concentrations of petroleum hydrocarbons; however, their ability to settle and metamorphose was adversely affected at environmentally relevant concentrations, and these effects were paralleled by marked changes in sponge gene expression and preceded by disruption of the symbiotic microbiome. Given the ecological importance of sponges, uncontrolled hydrocarbon releases from shipping accidents or production could affect sponge recruitment, which would have concomitant consequences for reef ecosystem function.

## INTRODUCTION

Tropical coral reefs are currently facing unprecedented declines due to global climate change and declining water quality (1). Natural hydrocarbon reservoirs are often found adjacent to coral reefs (2, 3), raising a unique conservation challenge as exploratory and extraction drilling are frequently undertaken in close proximity to these environmentally important biodiversity hot spots. Petroleum hydrocarbon exposures from shipping accidents (4, 5) and spills from coastal and offshore processing facilities can significantly impact coral reef communities over decadal time scales (6, 7). Two high-profile oil spills, the Montara well-head platform incident off northwest Australia (which released ∼4,500 m^3^ of medium crude oil into the Timor Sea) (8–10) and, shortly afterwards, the Macondo Deepwater Horizon incident (which released ∼780,000 m^3^ of light crude oil into the Gulf of Mexico) (11–14), emphasize the importance of understanding the effects of hydrocarbon spills and response interventions (e.g., application of chemical dispersants) on sessile reef invertebrates.

Marine sponges can occupy up to 80% of the available substrate and are ecologically important constituents of benthic environments as they provide habitat for a diverse array of epi- and endofauna, couple the benthic and pelagic zones by filtering large quantities of seawater, mediate biogeochemical fluxes, and facilitate consumption and release of nutrients (15–20). Sponges often host dense and diverse microbial communities which can comprise up to 35% of the host biomass and contribute to many aspects of the sponge’s physiology and ecology (21–23). Considering the functional importance of the microbiome for host health, sponges are often described as “holobionts,” indicating an interdependent consortium comprising the sponge host and the associated bacteria, archaea, unicellular algae, fungi, and viruses (24). In determining the sensitivity of marine sponges to environmental stressors, such as hydrocarbons, it is therefore necessary to consider the response of both the host and the symbiotic microbial community. To date, very little research has addressed how hydrocarbons and other petroleum products affect the sponge holobiont, particularly for early life history stages and processes (25–29).

Marine sponges often have decoupled life history stages, with the planktonic larvae of many species performing vertical migration to aid dispersal by optimizing exposure to water currents (30). This behavior may bring them into direct contact with water-soluble and entrained oil as well as with surface slicks following oil spills. Understanding the impact of hydrocarbon exposure on marine larvae is critical because the survival of early life history phases underpins reef recovery and resilience following disturbances (31, 32). A few field (5) and laboratory (29, 33–39) studies have described significant adverse effects of hydrocarbon exposure on the early life history stages of corals, with larval settlement generally considered to be one of the most sensitive early life history processes (29). Oil spill interventions often involve the application of large quantities of chemical dispersants (including surfactants) to promote oil solubility and reduce the impact of surface slicks (40). While dispersants have a lower toxicity than dissolved oil, they can increase the solubility of polycyclic aromatic hydrocarbons (PAHs) and therefore increase exposure to benthic and pelagic organisms (41). Despite the ecological importance of sponges, there is no available data on how they respond to dispersants, and only two studies have tested the impacts of oils or PAHs on sponge larvae, with contradictory results. While larvae of the encrusting sponge Crambe crambe were described as being sensitive to hydrocarbon exposure, with a nominal concentration of 0.5 μg liter^−1^ PAH mix (25) affecting metamorphosis, larvae of the demosponge Rhopaloeides odorabile were insensitive to condensate (liquid fraction from gas wells), with metamorphosis unaffected until dissolved total petroleum aromatic hydrocarbon (TPAH) concentrations exceeded 11,000 μg liter^−1^ (29).

Organisms cope with environmental stress by modifying their physiological functions and gene expression patterns to achieve cellular homeostasis (42). Although researchers have explored shifts in sponge gene expression in response to thermal stress (43–48), heavy metals (49, 50), and polychlorinated biphenyls (51), the molecular-level stress response of sponges to hydrocarbons has never been assessed. Similarly, a considerable body of research has evaluated how the sponge microbiome responds to various stressors, including temperature (52–54), carbonate chemistry (55), nutrients (56, 57), heavy metals (58–60), and sediments (61–64), but no studies have assessed how sponge symbionts respond to hydrocarbons. Interestingly, while many of these sponge microbiome studies report microbial community shifts with declining host health, others report remarkably stable microbial communities irrespective of host health or stressor level, indicating that the environmental sensitivity of sponge microbiomes is highly species and stressor specific. In addition, metaproteomic research has shown that while the genomic composition of the sponge microbiome may stay relatively stable upon initial exposure to environmental stress, expression of important symbiotic functions can be immediately affected, and this dysbiosis likely contributes to the overall host stress response (65).

The toxicity of crude oils extracted from the Northwest Shelf of Australia has been assessed for several temperate and tropical species (34, 66), yet the toxicity to sessile tropical reef sponges is unknown. To comprehensively explore the impacts of oil pollution on the larval sponge holobiont, we examined the acute toxicity of various concentrations of (i) water-accommodated fractions (WAFs) of crude oil, (ii) chemically enhanced WAFs (CWAFs) of crude oil, and (iii) dispersant to larvae of the abundant reef sponge Rhopaloeides odorabile. To quantify the holobiont stress response, we applied a multifaceted approach integrating standard ecotoxicological testing, larval settlement assays, multiplexed reverse transcription-quantitative PCR (mRT-qPCR) host gene expression analysis, and community profiling of the symbiotic microbial community. Identifying sensitive biological indicators for sponge stress responses to hydrocarbons will contribute to improving risk assessments and informing oil spill responses for the oil and gas industry, regulators, and spill responders.

## RESULTS

To determine the larval sponge holobiont response to hydrocarbon exposure, a broad suite of response variables were measured, including survival, metamorphosis, host gene expression, and microbiome composition. The sensitivity of each of these parameters is summarized in Table 1. For ease of reference, the sensitivity of each of the endpoints is reported throughout the text as percent WAF or percent CWAF and total PAH (ΣPAH). The respective total petroleum hydrocarbons (TPH) and dispersant Corexit EC9500A concentrations can be found in Table 1.

**TABLE 1:** Summary of response variables for each petroleum product treatment concentration^*a*^

Treatment and concn (%)	ΣPAH (μg/liter)	TPH (μg/liter)	Corexit EC9500A (mg/liter)^*b*^	Survival (%)^*c*^	Metamorphosis (%)^*c*^	Gene expression	Sponge microbiome
WAF							
0	0	0	ND	100	31 ± 6	ND	ND
0.8	0.86	32.5	ND	100	25 ± 6	ND	ND
1.6	1.7	65.0	ND	100	28 ± 5	X	✓
3.1	3.3	126	ND	100	24 ± 8	ND	ND
6.3	6.8	256	ND	100	28 ± 2	ND	ND
13	13.9	528	ND	100	6.7 ± 3.9	ND	ND
25	26.8	1,015	ND	99 ± 1	8.0 ± 3.3	✓	✓
50	53.6	2,030	ND	100	1.3 ± 1.1	ND	ND
75	80.4	3,045	ND	100	4.0 ± 1.9	ND	ND
100	107.2	4,060	ND	100	2.7 ± 1.1	✓	✓
CWAF							
0	0	0	0	100	31 ± 6		
0.8	0.58	273.6	19	100	2.7 ± 2.2	ND	ND
1.6	1.2	547.2	38	100	9.3 ± 4.4	X	✓
3.1	2.2	1,060	74	100	1.3 ± 1.1	ND	ND
6.3	4.6	2,155	149	100	4.0 ± 1.9	ND	ND
13	9.4	4,446	308	100	2.7 ± 1.1	ND	ND
25	18.1	8,550	593	100	1.3 ± 1.1	✓	✓
50	36.2	17,100	1,186	0	0	✓^≠^	X
75	54.2	25,650	1,779	0	0	ND	ND
100	72.3	34,200	2,373	0	0	ND	ND
Corexit							
0	ND	ND	0	100	31 ± 6	ND	ND
0.8	ND	ND	19	100	82.7 ± 4.4	X	ND
1.6	ND	ND	38	100	5.3 ± 1.1	✓^≠^	ND
3.1	ND	ND	74	4.0 ± 3.3	0	ND	ND
6.3	ND	ND	149	0	0	ND	ND
Other^*d*^	ND	ND	≥308	0	0	ND	ND

a Petroleum hydrocarbon analysis for total polycyclic aromatic hydrocarbons (ΣPAH) and total petroleum hydrocarbon (TPH) analysis can be found in Table S1 in the supplemental material. Light gray shading and X denote no significant difference; dark gray shading and a check mark (✓) denote a significant difference relative to levels in the control samples of the corresponding treatment (*P* < 0.05). ND, not done; ≠, gene expression change observed at 2 h, with no samples remaining to test at 24 h.

b Nominal concentration.

c Survival and metamorphosis were scored after 48 h (mean ± standard error).

d Concentrations of 13, 25, 50, 75, and 100%.

10.1128/mSystems.00743-19.2 TABLE S1 SIMPER analysis of the genes from the control and the 25% WAF and CWAF exposures. Download Table S1, DOCX file, 0.02 MB. © Crown copyright 2019. 2019 Crown This content is distributed under the terms of the Creative Commons Attribution 4.0 International license.

### Larval survival and metamorphosis.

**FIG 1:**
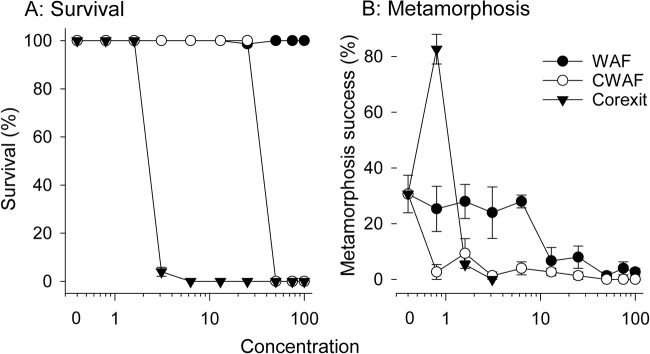
Mean survival (A) and metamorphosis success (B) of sponge larvae exposed to WAFs, CWAsF, and Corexit EC9500A after 48 h versus concentrations of the treatments in percentages (*n* = 3 replicates per concentration ± standard error). Results are presented relative to percent treatment solution as the three solutions were prepared identically (corresponding ΣPAH, TPH, and Corexit EC9500A concentrations for each dilution are listed in Table 1).

Larval survival was 100% in control samples and remained unaffected at all WAF concentrations including 100% (Table 1; Fig. 1A). In contrast, all larvae exposed to ≥50% CWAF were killed, as were all larvae exposed to ≥3.1% Corexit EC9500A (Table 1; Fig. 1A). Due to sharp drops from 100% to 0% survival for both CWAF and Corexit EC9500A treatments, 50% lethal concentration (LC_50_) values could not be calculated. The no-observed-effect concentration (NOEC) and lowest-observed-effect concentration (LOEC) for each treatment are reported in Table 2.

**TABLE 2:** Concentrations of total PAHs and dispersant with effects on survival and metamorphosis

Response variable and parameter^*a*^	WAF ΣPAH		CWAF ΣPAH		Corexit EC9500A	
	Concn (μg/liter)	Treatment (%)^*d*^	Concn (μg/liter)	Treatment (%)^*d*^	Concn (mg/liter)	WAF treatment (%)
Survival						
LOEC			18.1	25	38	1.6
NOEC	>107	100	36.2	50	19	0.8
Metamorphosis						
LOEC	14	13	0.58	0.8	38	1.6
NOEC	6.8	6.3	<0.1		19	0.8
EC_50_	12	6.3–13^*b*^	NA^*c*^		NA	

a Lowest-observed-effect concentration (LOEC) and no-observed-effect concentration (NOEC) for ΣPAH were calculated from one-way ANOVA (P < 0.01). EC_50_ settlement in sponge larvae was calculated from four-parameter logistic models (see Fig. S1 in the supplemental material).

b Values represent the 95% confidence interval.

c NA, not available. The EC_50_ could not be calculated due to limited data points on the slopes of dose-response curves.

d Corresponding TPH concentrations can be read from Table 1.

10.1128/mSystems.00743-19.1 FIG S1 Metamorphosis of sponge larvae (percent relative to 0% WAF controls) for ΣPAHs fitted to four-parameter logistic curve. Download FIG S1, PDF file, 0.1 MB. © Crown copyright 2019. 2019 Crown This content is distributed under the terms of the Creative Commons Attribution 4.0 International license.

**FIG 2:**
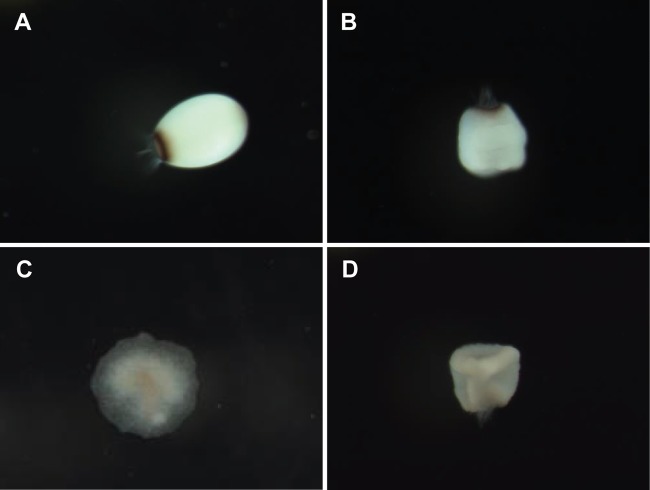
Planktonic larvae in control (A) and 25% CWAF (B) treatments after 24 h of treatment exposure. Larvae under control conditions successfully settle and metamorphose (C), whereas larvae treated with 25% CWAF were deformed and did not successfully metamorphose (D). Approximate larval length is 270 ± 4.17 μm (113).

Metamorphosis of R. odorabile larvae was defined as the point at which the planktonic larvae (Fig. 2A) attached to the surface and underwent flattening of the entire body to form a disc-like morphology, with the center showing the remnants of the posterior larval pole (Fig. 2C) (30). Larval metamorphosis was 31% ± 6% in control treatments (Fig. 1B). The 13% WAF treatment caused significant (*P* < 0.01; analysis of variance [ANOVA], *F*_9, 33_ = 4.2) reductions in successful metamorphosis to 6.7% (Fig. 1B and Table 1). The 50% effective concentration (EC_50_) value for ΣPAHs in the WAF was 12 μg liter^−1^ (95% confidence interval [CI], 6.8 to 18 μg liter^−1^) (Table 2; see also Fig. S1 in the supplemental material). Larval metamorphosis was significantly reduced at all CWAF concentrations of ≥0.8% (*P* < 0.01; ANOVA, *F*_9, 33_ = 6.4) but the EC_50_ values for CWAF could not be calculated as there were limited data between minimum and maximum inhibition levels (Fig. S1). Larvae exposed to the higher CWAF concentrations mutated into irregular shapes and did not successfully metamorphose (Fig. 2B and D). The addition of Corexit EC9500A alone significantly inhibited larval metamorphosis to 5% at 38 mg liter^−1^ (*P* < 0.01; ANOVA, *F*_9, 33_ = 33.3), and this decreased to zero at higher Corexit EC9500A concentrations (Table 1), but interestingly metamorphosis was stimulated to 83% at 19 mg liter^−1^ (Fig. 1B; Table 1).

### Host gene expression.

**FIG 3:**
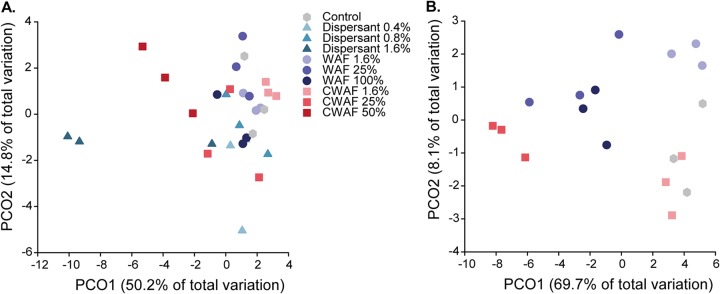
PCO based on the Bray-Curtis similarity of gene expression values from 26 selected host genes after 2 h (A) and 24 h (B).

Larval gene expression was significantly affected by petroleum hydrocarbons after only 2 h exposure (permutational multivariate analysis of variance [PERMANOVA], pseudo-*F*_9,20_ = 4.31, *P* = 0.001) (Fig. 3A). The ordination demonstrates two clear patterns: first the separation of the 1.6% Corexit EC9500A (38 mg liter^−1^) treatment from all other samples and, second, a notable separation of samples in the 25% (18.1 μg liter^−1^ ΣPAH) and 50% (36.2 μg liter^−1^ ΣPAH) CWAF treatments from the controls (Fig. 3A). After 24 h, larvae from the 1.6% (1.7 μg liter^−1^ ΣPAH) WAF and 1.6% (1.2 μg liter^−1^ ΣPAH) CWAF treatments were not significantly different from those of the controls (*P* > 0.05); however, a significant difference was detected at 25% WAF (26.8 μg liter^−1^ ΣPAH; Monte Carlo *P* value [*P*(MC) = 0.012]) and 25% CWAF [18.1 μg l ^−1^ ΣPAH; *P*(MC) = 0.001], also clearly separated in the ordination (Fig. 3B). Similarity percentage (SIMPER) analysis of samples from the 24-h exposure revealed that increased expression of heat shock protein 70 (HSP70) (29.56%), actin-related protein 2/3 (ARP2/3) complex (6.97%), profilin (6.13%), actin (5.57%), ferritin (5.57%), and HSP90 (5.26%) contributed most to the dissimilarity in expression profiles between samples in the control and 25% WAF (26.8 μg liter^−1^ ΣPAH) treatments (Table S1). Increased expression of HSP70 (26.38%), polyubiquitin (11.35%), ferritin (10.11%), profilin (6.92%), and HSP90 (6.82%) also contributed most to the dissimilarity in gene expression profiles between samples in the control and 25% CWAF (18.1 μg liter^−1^ ΣPAH) treatments after 24 h (Table S1). No significant differences in gene expression levels were evident between 25% WAF (26.8 μg liter^−1^ ΣPAH) and 100% WAF (107.2 μg liter^−1^ ΣPAH) (*P* > 0.05).

### Microbial community analysis.

**FIG 4:**
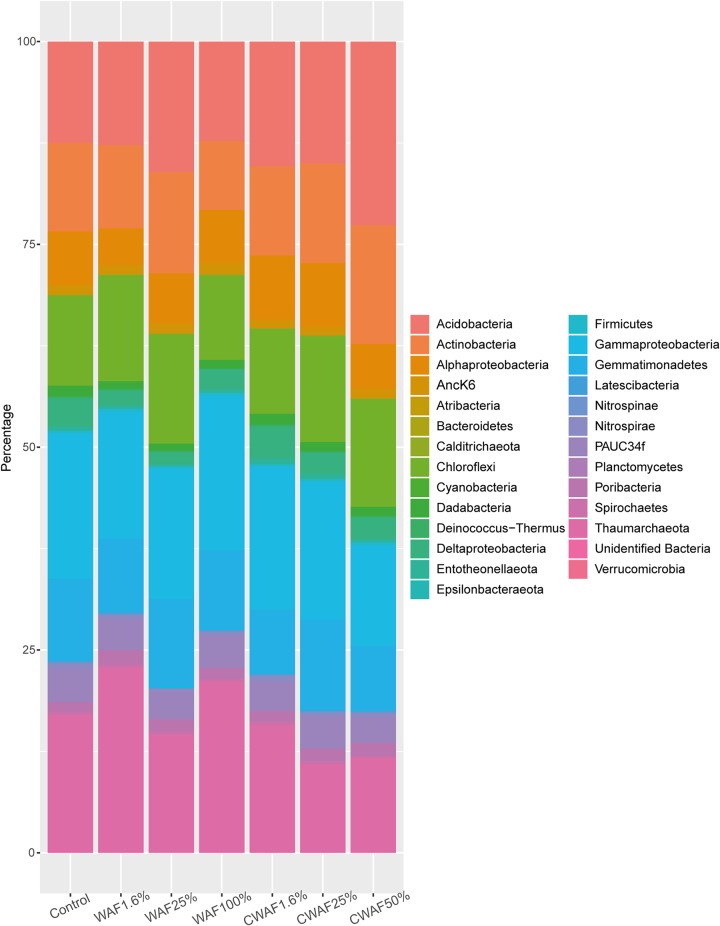
Stacked bar chart depicting the relative abundance of each bacterial phyla, plus class for *Proteobacteria*, associated with each treatment.

**FIG 5:**
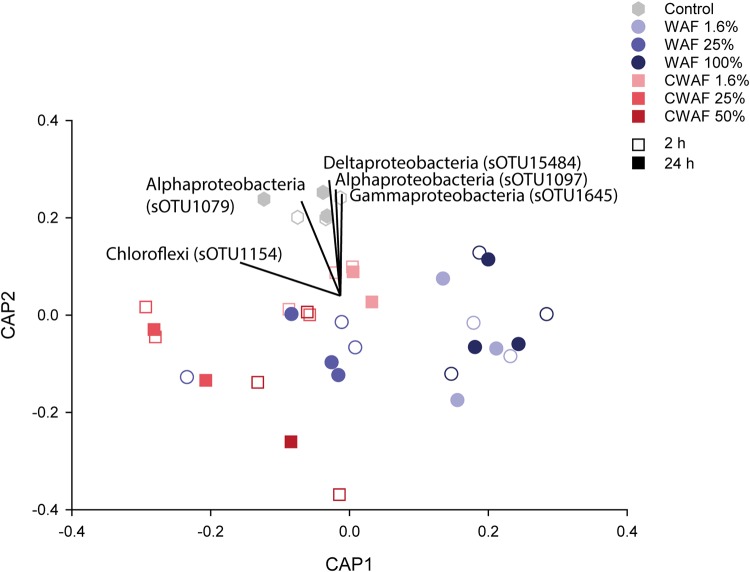
CAP analysis based on Bray-Curtis similarity of the OTUs derived from 16S rRNA gene sequencing of the Rhopaloeides odorabile larval microbiome from each treatment after 2 and 24 h.

**FIG 6:**
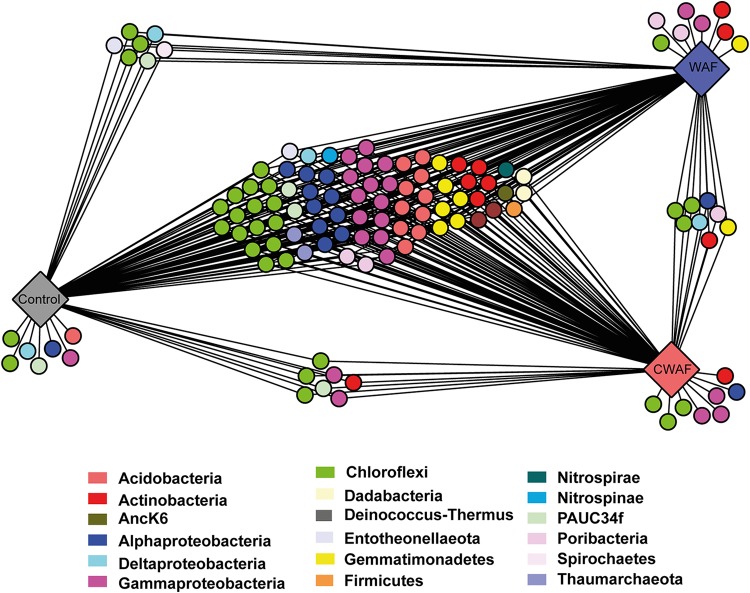
Cytoscape networks created using the 100 most abundant OTUs from each treatment.

The R. odorabile microbiome is dominated by *Gammaproteobacteria*, *Thaumarchaea*, *Acidobacteria*, *Gemmatimonadetes*, *Chloroflexi*, PAUC34f, and *Actinobacteria* (Fig. 4). The microbiome was significantly affected by hydrocarbon treatment (PERMANOVA, pseudo-*F*_6_ = 1.655, *P* = 0.0438) (Fig. 4 and 5), with the microbial communities of sponge larvae exposed to WAF treatments of 1.6% (*P* = 0.0378), 25% (*P* = 0.0325), and 100% (*P* = 0.0258) all significantly different from those of the control samples. In contrast, the microbiome of CWAF-exposed larvae was only significantly different from that of the controls at 1.6% (*P* = 0.0171) and 25% (*P* = 0.0383) CWAF. While samples exposed to 50% CWAF were not significantly different, they clustered further from control samples in the ordination than the other two CWAF treatments (Fig. 5). The nonsignificant result likely reflects lower replication with this treatment (*n* = 4) (Table S2). A significant difference between time points was also observed (PERMANOVA, pseudo-*F*_6_ = 2.9448, *P* = 0.01), but no interaction between treatment and time was identified (PERMANOVA, pseudo-*F*_6_ = 0.9951, *P* = 0.1734), with treatment differences more distinct than those of time (Fig. 5). A previously described R. odorabile thaumarchaeal symbiont (sub-operational taxonomic unit 137 [sOTU137]) (67) also significantly decreased in abundance across all hydrocarbon treatments (ANOVA, *F*_6_ = 2.45, *P* = 0.04). A decrease in the relative abundances of *Thaumarchaea* was evident in sponges exposed to treatments of 25% CWAF and above, and a decrease in *Gammaproteobacteria* was detected at 50% CWAF (Fig. 4). In contrast, an increase in the relative abundance of *Acidobacteria* was evident in the microbiome of sponges exposed to the 50% CWAF treatment (Fig. 4). To identify specific microbial sOTUs primarily responsible for driving differences in community composition between control and WAF- and CWAF-treated samples, Cytoscape network analysis was performed using the 100 most abundant sOTUs in each treatment data set (i.e., control, WAF, and CWAF). While many of the dominant sOTUs were present across all treatments, seven OTUs were exclusively present in control samples, eight OTUs were exclusive to samples in the WAF treatment, and eight were exclusive to samples in the CWAF treatment, with an additional eight OTUs being shared between the WAF- and CWAF-treated samples but absent from the controls (Fig. 6; see Table S3 for sOTU details). Treatment-specific OTUs spanned multiple bacterial phyla and classes (Fig. 6; Table S3).

10.1128/mSystems.00743-19.3 TABLE S2 EMP sample IDs for the WAF and CWAF exposure experiments. Download Table S2, DOCX file, 0.02 MB. © Crown copyright 2019. 2019 Crown This content is distributed under the terms of the Creative Commons Attribution 4.0 International license.

10.1128/mSystems.00743-19.4 TABLE S3 Taxonomic assignment of the most abundant sOTUs that were exclusive to a treatment. Symbionts of sponges are indicated by an asterisk (*) whereas symbionts of coral species are indicated by a plus sign (+). Download Table S3, DOCX file, 0.02 MB. © Crown copyright 2019. 2019 Crown This content is distributed under the terms of the Creative Commons Attribution 4.0 International license.

## DISCUSSION

### General.

Sponges perform a range of important functional roles in marine systems (15), particularly on coral reefs where they process large volumes of seawater and efficiently remove the particulate and dissolved organic carbon (68, 69). The current study showed that R. odorabile larvae can survive high concentrations of petroleum hydrocarbons, but their ability to undergo successful settlement, crucial for recruitment, is affected at moderate concentrations of PAHs. This effect was exacerbated by the addition of the dispersant Corexit EC9500A. Effects on host gene expression and the associated microbiome were evident at sublethal concentrations of PAHs, in both the presence and absence of dispersant, providing valuable insights into stress response pathways. Considering the sensitivity of the symbiotic microbial community, assessment of the microbiome represents a promising indicator for monitoring sublethal stress responses in this sponge species.

### Larval survival and settlement.

Although concentrations of PAHs are low in pristine coral reef ecosystems (70), the concentrations found in tropical and subtropical marine environments can be as high as 34.4 μg liter^−1^ in areas with no obvious signs of contamination (71–73). However, after large-scale accidental releases, such as the Deep Water Horizon spill, PAH concentrations reached ≥189 μg liter^−1^ (74), and even higher levels have been detected following bilge water discharges (e.g., 13,700 μg liter^−1^) (72). While R. odorabile larvae in this study were able to survive high concentrations of petroleum hydrocarbons, they lost the ability to settle and metamorphose at environmentally relevant concentrations (e.g., 13.9 to 26.8 μg liter^−1^).

The high tolerance of R. odorabile larvae to light crude WAFs from the Northwest Shelf of Australia is consistent with previous work showing high survival of the same species to WAFs of condensate (derived from a lighter Western Australian condensate) (29). Larval metamorphosis was more sensitive to the light crude oil in the present study (NOEC = 14 μg liter^−1^ ΣPAH) than to condensate exposures (NOEC = 121 μg liter^−1^ ΣPAH). These concentrations of PAHs (≥189 μg liter^−1^) were less than the concentrations identified in seawater following the Deep Water Horizon spill (74). However, comparing sensitivities of marine species to petroleum hydrocarbons between studies is notoriously difficult due to differences in exposure methodologies and in the ways in which hydrocarbon concentrations are measured and expressed (75, 76). For instance, the discrepancy in sensitivities between the two R. odorabile studies could be attributed to the WAFs from the current study having been prepared with more energy (a greater vortex), which would result in more whole-oil droplets in suspension (entrained oil, measured as TPH). These higher-energy WAF preparations are generally considered more toxic than lower-energy WAF preparations (77). The only other study to examine effects of PAHs on sponges found inhibition of metamorphosis of Crambe crambe larvae at only 0.5 μg liter^−1^ ΣPAH (25). The sensitivity of R. odorabile is more consistent with the sensitivity of coral larvae to condensate/light crude (29, 33), fuel oil (39), and individual PAHs (78); however, the disparate sensitivities of the only two sponge species analyzed to date highlight the need for standardized and comparative studies to establish relative species sensitivities of sponge larvae to oil pollution.

Chemical dispersion of the light crude oil by the dispersant Corexit EC9500A markedly increased the apparent toxicity of the treatments, causing total larval mortality and reduced metamorphosis at 50% and 13% CWAFs, respectively (compared with >100% and 50% for WAFs). This increase in toxicity is likely due to changes in the chemical composition of the test solutions, with CWAF containing >10-fold more TPHs than WAF, as well as the Corexit EC9500A itself. The lowest CWAF concentration 0.8% (0.58 μg liter^−1^ ΣPAH; 19 mg liter^−1^ Corexit) caused significant inhibition of metamorphosis, while metamorphosis was reduced at only 1.6% (38 mg liter^−1^) Corexit EC9500A solution alone, indicating that the combined effect of oil and dispersant was responsible for this higher larval sensitivity. Similar increases in toxicity of oil in the presence of dispersant have been observed for other marine species, including corals (34, 79–81). Sponge larval metamorphosis had a similar sensitivity to Corexit E9500A (LOEC = 38 mg liter^−1^) as larvae from multiple coral species (LOEC of 5 to 70 mg liter^−1^) (33, 82–84) (EC_50_ = 14 mg liter^−1^) (85). Intriguingly, the lowest exposure of Corexit EC9500A (19 mg liter^−1^) caused a large increase in settlement and metamorphosis (Table 1 and Fig. 1B). The most parsimonious explanation for this result is that, at this concentration, the dispersant mimics an external chemical inducer or internal signaling molecule that initiates metamorphosis. However, it may also be a sublethal stress response as thermal stress has been shown to increase settlement in this species (86). This type of response has not been reported for coral larvae over a wider range of exposures to five dispersants, including Corexit EC9500A (85), and further investigation is warranted as control of larval settlement in sponges may be useful for *in vitro* studies or reef restoration practices.

### Gene expression.

Larval gene expression patterns were significantly affected at 26.8 μg liter^−1^ ΣPAH in the WAF treatment and at 18.1 μg liter^−1^ ΣPAH in the CWAF treatment. Host gene expression was disrupted by WAF and CWAF concentrations 2- to 4-fold lower than those causing larval mortality. Heat shock protein 70 (HSP70) contributed most to the differences between the control and the WAF and CWAF treatments, and HSP70 and HSP90 combined were responsible for 35% of the variation in expression, a stress response consistent with what has been observed for this species following exposure to elevated temperature (45). A similar molecular-level response has also been observed in corals, with increased expression of both HSP70 and HSP90 in Acropora tenuis larvae exposed to anthracene (78). Similarly, HSP70 was significantly upregulated in the coral Pocillopora damicornis when it was exposed to WAFs (87); and although expression levels were not quantified, HSP70 was identified via RT-PCR in the adult coral Stylophora pistillata exposed to five different WAF concentrations yet was undetectable in the control treatment (88). Other toxicants, such as heavy metals, induce a similar cellular stress response in reef taxa, with an upregulation of HSP70 identified in corals (89), ascidians (90), and sponges (91). Here, we observed changes in host gene expression profiles at sublethal concentrations of both WAFs and CWAFs. Given the sensitivity of HSP70 in multiple taxa exposed to various contaminants (78, 87), this gene represents a strong general bioindicator candidate for use to detect sublethal stress responses in marine species exposed to oil and pollution generally.

### Sponge microbiome.

The R. odorabile larval microbiome was highly sensitive to hydrocarbon exposure, with a shift in the microbiome occurring at concentrations as low as 1.7 μg liter^−1^ ΣPAH in the WAF treatment and 1.2 μg liter^−1^ ΣPAH in the CWAF treatment. Sponge symbionts undertake a broad range of metabolic functions, including carbon, nitrogen, and sulfur metabolism, vitamin synthesis, production of bioactive metabolites, and nutrient transport (92–94); hence, microbial shifts or loss of key symbionts can have adverse impacts on the holobiont (52, 65, 95). Of particular interest for R. odorabile larvae exposed to hydrocarbons was the significant reduction in a putatively ammonia-oxidizing thaumarchaeal symbiont (67). The sensitivity of the R. odorabile thaumarchaeal symbiont is consistent with recent analyses showing that ammonia-oxidizing archaea are ∼1,000 times more sensitive to hydrocarbon contamination than heterotrophic bacteria (96). However, it could also be that this symbiont is particularly sensitive to environmental perturbation as previous research has demonstrated that it is highly sensitive to heavy metal contamination (60). Several microbial OTUs were identified as being exclusive to WAF (*n* = 8) or CWAF (*n* = 8) treatments, and these OTUs spanned multiple taxa, including *Gammaproteobacteria*, *Alphaproteobacteria*, *Chloroflexi*, *Gemmatimonadetes*, *Poribacteria*, and *Actinobacteria* (see Table S3 in the supplemental material). Interestingly, OTUs exclusive to WAF or CWAF treatments shared highest percent similarity to other sponge- or coral-associated bacteria. However, despite being among the 100 most abundant OTUs, taxa that were exclusive to the WAF and CWAF treatments comprised <1% of the total microbial community. It is likely that these OTUs are exceptionally rare (and therefore undetectable) in the sponge microbiome under control conditions but become selected for in the WAF and CWAF treatments. Alternatively, these novel microorganisms may have been acquired from the surrounding seawater as a low abundance of sponge-specific microbes has been previously detected within the rare seawater biosphere (97). Future studies should employ metagenomic approaches to determine whether these symbionts have the genomic potential to degrade hydrocarbons as previous studies of seawater (98–100), sediments (101–103), sand (104), biofilms (98), phytoplankton (105), mussels (106), sponges (106), and corals (107) have all shown increased relative abundances of putative hydrocarbon degraders following oil exposure.

Several recent studies have highlighted the potential for microorganisms to act as sensitive markers for environmental disturbance in reef ecosystems (reviewed in reference 108). In particular, sponge symbionts have been described as sublethal stress indicators for elevated seawater temperature (52, 53, 65) and copper contamination (60). This high environmental sensitivity supports the diagnostic value of the sponge microbiome and highlights how coral reef monitoring initiatives could be enhanced by incorporating assessments of sponge symbionts. The coral microbiome has also been shown to shift after exposure to crude oil, including higher relative abundances of putative hydrocarbon degraders such as *Pseudomonas*, *Pseudoalteromonas*, and *Alteromonas* versus a dominance of *Vibrio* in corals not exposed to oil (109). However, given Santos et al. used a longer exposure time (4 to 16 weeks) and did not perform chemical analysis, it remains unknown whether the coral microbiome is as responsive to WAFs as the sponge-larval microbiome.

Larval R. odorabile can survive high concentrations of WAFs; however, a loss of critical biological function is detected at spill-relevant ΣPAH concentrations, as evidenced by adverse effects on metamorphosis, settlement, host gene expression, and the microbiome. Clearly, exposure to petroleum hydrocarbons from accidental releases or spills has the potential to negatively impact sponge recruitment to adult populations, which can have adverse consequences for the ecology of reef systems. The identification of toxic thresholds (NOEC = 6.9 μg liter^−1^ ΣPAH) and effective concentrations (EC_50_ = 12 μg liter^−1^ ΣPAH) for sponge larval settlement for light crude oil adds to the very limited data available on coral reef-associated taxa. This study also revealed changes in sponge larval gene expression upon PAH exposure, particularly, increased expression of the HSP70 and HSP90 genes, which is consistent with reports for other marine species (78, 87). Importantly, the sponge microbiome proved to be the most sensitive indicator of sublethal stress following exposure to petroleum hydrocarbons and Corexit EC9500A. To better understand the consequences of this microbial dysbiosis (such as the reduced relative abundance of the dominant thaumarchaeal symbiont in PAH exposed sponges), future research should employ metagenomic and metatranscriptomic approaches to validate the link between disruption of key microbial pathways and host health. Finally, the clearly distinct microbial communities that develop in sponge larvae from the WAF, CWAF, and Corexit EC9500A treatments highlight the diagnostic utility of the R. odorabile microbiome as a sensitive *in situ* marker for exposure to hydrocarbon contamination. Monitoring of the R. odorabile microbiome has the potential to provide regulators and industry with an early indication of oil contamination on coral reefs.

## MATERIALS AND METHODS

### Preparation of WAFs and CWAFs.

A sample of light crude oil (36.1° American Petroleum Institute [API] gravity) from Barrow Island (northwest Western Australia) was provided by Chevron Australia, and the dispersant Corexit EC9500A was provided by the Australian Maritime Safety Authority. Water-accommodated fractions (WAFs) and chemically enhanced water-accommodated fractions (CWAFs) were prepared from the crude oil as previously described (110, 111). Briefly, the WAF was prepared by adding 1,600 ml of filtered (0.45-μm pore size) seawater (36 practical salinity units [PSU], pH 8.1) to a solvent-rinsed 2-liter glass aspirator bottle and mixed using a magnetic stirrer to generate a 20 to 25% vortex. Crude oil (40 ml) was subsequently added to the center of the vortex to achieve a concentration of 25 ml liter^−1^, the aspirator was loosely capped, and fluids were mixed for 18 h in darkness. To prepare CWAF, 4 ml of the dispersant Corexit EC9500A (1:10 dispersant/oil) was gently added to the top of the vortexing mixture described above and allowed to mix for 18 h (112). The WAFs and CWAFs were allowed to settle for 6 h before immediate water sampling for chemical analyses and applications in the larval assays. Dilutions of the 100% WAF and CWAF (100, 75, 50, 25, 12.5, 6.25, 3.13, 1.56, 0.78, and 0 % [vol/vol]) were prepared using filtered (0.45-μm pore size) seawater to mimic dilution in the water column (112). A separate solution of Corexit EC9500A was prepared in the same way by applying 4 ml of dispersant to 1,600 ml of filtered seawater, mixing, settling, and diluting as described above. Total petroleum hydrocarbons were analyzed by gas chromatography flame ionization detection (Queensland Government Forensic and Scientific Services [QHFSS] method 16308), and PAHs were analyzed by gas chromatography-mass spectrometry (QHFSS method 16647) at the National Association of Testing Authorities (NATA)-accredited Queensland Government Forensic and Scientific Services (Archerfield, Queensland, Australia). The 100% WAF and 100% CWAF contained 107 and 72 μg liter^−1^ total polycyclic aromatic hydrocarbons (ΣPAHs), respectively, and the total petroleum hydrocarbon (TPH) concentrations in the 100% WAF and the 100% CWAF were 1 and 2 orders of magnitude higher than the concentration of ΣPAHs, respectively (Table 1; see Table S4 in the supplemental material), indicating the presence of oil droplets in both preparations.

10.1128/mSystems.00743-19.5 TABLE S4 Concentrations of TPH and ΣPAH in the 100% WAF and CWAF of Barrow Island oil used in the current study. RL is the reporting limit. Download Table S4, DOCX file, 0.01 MB. © Crown copyright 2019. 2019 Crown This content is distributed under the terms of the Creative Commons Attribution 4.0 International license.

### Sponge collection and larval culture.

Rhopaloeides odorabile is a common gonochoristic Great Barrier Reef (GBR) sponge that broods tufted parenchymella larvae that are released during the Austral summer (113). Seven female sponges were collected from Davies Reef, central GBR, Australia (18°50.558′S, 147°37.618′E) and transported to the Australian Institute of Marine Science (AIMS). Sponges were maintained in flowthrough aquaria which allowed the controlled collection of larvae over several hours during their afternoon release. Larvae were collected using larval traps according to established methods (30, 114) and were pooled prior to being used in experimental assays.

### Larval settlement assays.

Static WAF and CWAF exposures were conducted in 7-ml glass vials made up to 6.5 ml with 10 dilutions of either WAF, CWAF, or Corexit EC9500A and containing 25 larvae. Three replicate vials were used for each of the treatment concentrations. Vials were sealed with caps leaving an ∼0.5-ml headspace that enabled oxygen exchange (O_2_ concentrations maintained at >7.5 mg liter^−1^ over the 24-h exposure). Vials were transferred to an incubator shaker with 40 μE of light over a 12-h/12-h cycle at ∼60 rpm to maintain gentle water movement. Vials were removed after 24 h of exposure, and the larvae and treatment solutions from individual vials were transferred directly into individual six-well cell culture plates (12 ml; Nunc, NY, USA) that had been immersed in flowthrough aquaria for 48 h to develop an early microbial biofilm required for successful settlement (115). Metamorphosis was assessed after 48 h and scored as positive if larvae had firmly attached to the surface and undergone flattening of the body to form a disc-like morphology, with the center showing the remnants of the posterior larval pole (Fig. 2C) (30).

Additional experiments were completed to examine changes in host gene expression and the symbiotic microbial community following exposure to hydrocarbon treatments during the larval swimming phase. This series of exposures included a control and three WAF/CWAF treatment dilutions (100%, 25%, and 1.6%), with three replicate vials maintained for each concentration. In addition, due to insufficient larval numbers, microbial assays did not contain the Corexit EC9500A treatment. Experimental hydrocarbon treatments were prepared, and treatment exposures were conducted, according to the same procedures outlined above, excluding the settlement assays. Gene expression and microbiome changes were assessed 2 h and 24 h after treatment exposure. At the end of each exposure period, larvae were removed from the treatments, rinsed in filtered seawater, immersed in liquid nitrogen, and stored at –80°C.

### Host mRT-qPCR analysis.

To investigate the expression profiles of 26 selected host genes in larvae exposed to three concentrations of WAF, CWAF, and Corexit EC9500A, we developed a multiplexed reverse transcription-quantitative PCR (mRT-qPCR) assay using a GenomeLab GeXP Genetic Analysis System (Beckman Coulter, Fullerton, CA). Experiments were conducted on pooled larvae for each treatment replicate, as previously described (45). Briefly, this method allows the sensitive and simultaneous detection of target genes in multiplexed reactions, with cDNA synthesis performed with target-specific primers and subsequent amplification with universal primers, removing the documented bias of PCR efficiency variation between genes. The set of 26 genes were selected based on their known or putative roles in the cell stress response and cellular homeostasis-related processes as previously described (44) (Table S5). Kanamycin (Kan^r^) was used as an internal control. Following the procedures of Webster and colleagues (45), mRNA was extracted from all larval sponge samples using a Dynabeads oligo(dT) kit (Invitrogen). Integrity of the mRNA was measured using an ND-1000 spectrophotometer (NanoDrop Technologies) with ratios of 260 nm/280 nm between 1.8 and 2 as the criteria. mRNA was reverse transcribed into cDNA and PCR amplified in 20-μl reaction mixtures containing 4 μl of PCR buffer (5×), 4 μl of MgCl_2_ (25 mM), 0.7 μl of Thermo-Start DNA polymerase (ABgene), 8.7 μl of cDNA, and 2 μl of forward primer (200 nM). The PCR thermal cycling protocol included 10 min at 95°C followed by 35 cycles of 30 s at 94°C, 30 s at 55°C, and 1 min at 70°C. PCR products were analyzed on an automated capillary electrophoresis sequencer CEQ 8800 Genetic Analysis System (Beckman-Coulter). Electropherograms were inspected for erroneous amplification products with a GenomeLab 178 Genetic Analysis System, version 10.0.29, software, and reproducibility was assessed by overlaying graphs from independent runs. Automatic filters were created to exclude false signals due to shoulder peaks, high homology, or alternative transcripts. Filtered positive data were imported and binned following a range extension of 2 bp in GenomeLab eXpress Profiler software. Finally, an expression stability measure according to Vandesompele et al. (116) for each of the 26 genes of interest was established in the GeNorm VBA applet for Microsoft Excel, and all positive amplicons were normalized against the geometric mean of the most stable pair of reference genes (RGs) (YWHAY and YWHAZ) in Excel. The geometric mean was calculated by averaging the Kan^r^ normalized peak area of the RG pair, and peak areas of all other genes of interest were divided by this geometric mean. Gene expression data for both time points can be found in Data Set S1.

10.1128/mSystems.00743-19.6 TABLE S5 List of genes used in the multiplexed RT-qPCR assay. Data are from reference 45. Download Table S5, DOCX file, 0.01 MB. © Crown copyright 2019. 2019 Crown This content is distributed under the terms of the Creative Commons Attribution 4.0 International license.

10.1128/mSystems.00743-19.7 Data Set S1 Gene expression data from the mRT-qPCR assay from both time points used as input for the SIMPER analysis. Download Data Set S1, XLSX file, 0.03 MB. © Crown copyright 2019. 2019 Crown This content is distributed under the terms of the Creative Commons Attribution 4.0 International license.

### DNA extraction, sequencing, and processing for microbial community profiling.

Genomic DNA was extracted from pooled larvae using a PowerSoil high-throughput 96-well DNA isolation kit (MoBio Laboratories, Inc.), according to the manufacturer’s protocol. As part of the Earth Microbiome Project (EMP) (117), samples were sent to the University of Colorado, Boulder, CO, where 16S rRNA genes were PCR amplified and sequenced on an Illumina HiSeq 2500 platform using bacterial primers 515F/806R and standard protocols (118).

Quality-filtered, demultiplexed fastq sequences were denoised by collaborators at the sponge microbiome project using Deblur (119). Briefly, to create the deblurred BIOM table input, sequences were trimmed to 100 bp, and the number of minimum reads was 25. Taxonomy was added using Qiime, the Ribosomal Database Project (RDP) Classifier, and Greengenes, version 13.8 (120). Samples from the current study (Table S1) were extracted from the larger BIOM table, and sOTUs were reclassified using the SILVA database (version 132), using a minimum cutoff of 60% similarity. Singletons and doubletons, i.e., sOTUs formed by one or two sequences, respectively, across all samples, were removed from the data set. Several samples were removed from the analysis due to low numbers of sequence reads, resulting in <3 replicates per time point for some treatments (Table S2).

### Data analyses.

Inhibition of metamorphosis (inhibition percent relative to 0% WAF control) was calculated from treatment data as follows: inhibition (%) = 100 × [(% metamorphosis_control_ − % metamorphosis_treatment_)/% metamorphosis_control_]. The concentrations of PAHs and TPHs that inhibited 50% of metamorphosis (EC_50_) were calculated from concentration-response curves (four-parameter logistic models) fitted to the percent inhibition and from concentration data of each treatment using the program GraphPad Prism (version 6; San Diego, CA, USA). Analysis of variance (ANOVA) was performed to identify treatments which caused significant (*P* < 0.05) inhibition of metamorphosis in comparison to that of control treatments (NCSS, version 9; NCSS, Kaysville, UT).

Principal coordinate analysis (PCO) was used to visually compare larval gene expression patterns among treatments, and canonical analysis of principal coordinates (CAP) was used to visually compare microbial community patterns among treatments and time points. PERMANOVA, using 9,999 permutations, was used to test differences in both gene expression levels and microbial community structures between treatments. Samples from the two time points were combined for the microbial analysis due to the low replication levels with some treatments, with time included in the model. Where pairwise comparisons resulted in insufficient unique permutations, Monte Carlo *P* values were used. Similarity percentage (SIMPER) analysis was used to determine genes that contributed to differences in expression patterns and OTUs that contributed to differences in microbial community structure. The distribution of the 100 most abundant sOTUs across larval treatments was visualized using Cytoscape, version 3.2.1 (www.cytoscape.org) (121). To minimize the number of nodes in the Cytoscape network, 0 and 1.6% WAF treatments were pooled and assigned to the control group, and 25 and 100% WAF treatments were pooled and assigned to the WAF group. Given the increased toxicity of CWAFs, the control group was made up only of the 0% CWAF treatment, whereas the CWAF group was made up of the 1.6, 25, and 50% CWAF treatments combined. All statistical analyses were based on Bray-Curtis distances of square root-transformed data and were performed using PRIMER 6/PERMANOVA+, version 1.0.2 (Plymouth, United Kingdom).

### Data availability.

Gene expression data for both time points can be found in Data Set S1. Processed sequences and metadata are available at http://qiita.microbio.me/ under study identification number 10793, and the deblurred BIOM table can be accessed through the GigaScience repository (https://doi.org/10.5524/100332) using sample identification numbers from Table S2.
