# Supplementary material for: The Effects of Crude Oil and Dispersant on the Larval Sponge Holobiont
Source: mSystems. 2019 Dec 10;4(6):e00743-19. doi: 10.1128/mSystems.00743-19 (PMC6906743; doi:10.1128/mSystems.00743-19)
Supplement: TABLE S1 [file mSystems.00743-19-st001.docx]

|  | **Control** | **25% WAF** |  |  |  |  |
| --- | --- | --- | --- | --- | --- | --- |
|  | Av.Abund | Av.Abund | Av.Diss | Diss/SD | Contrib% | Cum.% |
| HSP70 | 0.63 | 1.37 | 2.56 | 8.47 | 29.56 | 29.56 |
| Actin related protein 2/3 complex subunit 4 | 0.65 | 0.82 | 0.6 | 5.36 | 6.97 | 36.53 |
| Profilin | 0.54 | 0.69 | 0.53 | 3.02 | 6.13 | 42.66 |
| Actin | 1.27 | 1.34 | 0.48 | 2.09 | 5.57 | 48.23 |
| Ferritin | 0.79 | 0.93 | 0.48 | 1.69 | 5.57 | 53.8 |
| HSP90 | 0.98 | 1.08 | 0.46 | 1.68 | 5.26 | 59.06 |
| Ribosomal protein S9 | 0.84 | 0.95 | 0.38 | 2.22 | 4.44 | 63.5 |
| Polyubiquitin | 0.67 | 0.78 | 0.37 | 1.44 | 4.32 | 67.82 |
| β-tubulin | 0.72 | 0.83 | 0.37 | 1.89 | 4.26 | 72.08 |
| Villin | 0.63 | 0.73 | 0.34 | 2.12 | 3.93 | 76.01 |
| Radial spoke protein | 0.66 | 0.72 | 0.26 | 1.24 | 2.96 | 78.96 |
| Cyclophilin | 1.11 | 1.15 | 0.22 | 1.77 | 2.59 | 81.55 |
| Glutathione-S-transferase | 0.17 | 0.23 | 0.21 | 2.25 | 2.41 | 83.97 |
| Ubiquitin conjugating enzyme | 0.34 | 0.31 | 0.2 | 2.2 | 2.3 | 86.27 |
| Calmodulin | 0.53 | 0.55 | 0.2 | 1.69 | 2.26 | 88.53 |
| Apoptosis linked gene 2 | 0.49 | 0.49 | 0.19 | 2.51 | 2.24 | 90.77 |

|  | **Control** | **25% CWAF** |  |  |  |  |
| --- | --- | --- | --- | --- | --- | --- |
|  | Av.Abund | Av.Abund | Av.Diss | Diss/SD | Contrib% | Cum.% |
| HSP70 | 0.63 | 1.59 | 3.17 | 14.65 | 26.38 | 26.38 |
| Polyubiquitin | 0.67 | 1.09 | 1.36 | 9.84 | 11.35 | 37.73 |
| Ferritin | 0.79 | 1.16 | 1.21 | 13.08 | 10.11 | 47.84 |
| Profilin | 0.54 | 0.79 | 0.83 | 4.38 | 6.92 | 54.76 |
| HSP90 | 0.98 | 1.23 | 0.82 | 2.37 | 6.82 | 61.58 |
| Actin related protein 2/3 complex subunit 4 | 0.65 | 0.82 | 0.57 | 7.71 | 4.77 | 66.35 |
| β-tubulin | 0.72 | 0.88 | 0.52 | 4.91 | 4.37 | 70.72 |
| Actin | 1.27 | 1.39 | 0.4 | 1.73 | 3.33 | 74.06 |
| Ribosomal protein S9 | 0.84 | 0.96 | 0.4 | 5.16 | 3.33 | 77.38 |
| Glutathione-S-transferase | 0.17 | 0.29 | 0.39 | 3.51 | 3.21 | 80.59 |
| Gelsolin | 0.53 | 0.63 | 0.33 | 3.37 | 2.71 | 83.31 |
| Cyclophilin | 1.11 | 1.18 | 0.27 | 1.61 | 2.28 | 85.58 |
| Radial spoke protein | 0.66 | 0.72 | 0.24 | 1.24 | 2.02 | 87.6 |
| Calmodulin | 0.53 | 0.6 | 0.23 | 1.4 | 1.9 | 89.5 |
| Ubiquitin conjugating enzyme | 0.34 | 0.4 | 0.2 | 1.95 | 1.68 | 91.18 |
