# Supplementary figures and images for: The Effects of Crude Oil and Dispersant on the Larval Sponge Holobiont
Source: mSystems. 2019 Dec 10;4(6):e00743-19. doi: 10.1128/mSystems.00743-19 (PMC6906743; doi:10.1128/mSystems.00743-19)

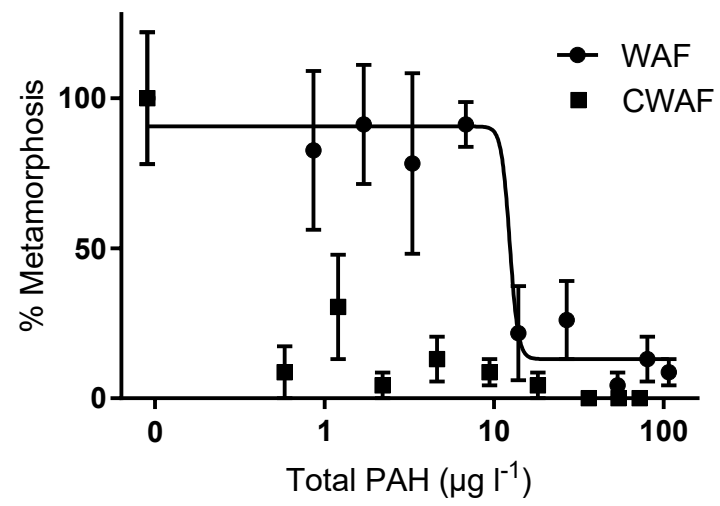

Supplement: FIG S1 [file mSystems.00743-19-sf001.pdf]
