# Supplementary material for: The Effects of Crude Oil and Dispersant on the Larval Sponge Holobiont
Source: mSystems. 2019 Dec 10;4(6):e00743-19. doi: 10.1128/mSystems.00743-19 (PMC6906743; doi:10.1128/mSystems.00743-19)
Supplement: TABLE S2 [file mSystems.00743-19-st002.docx]

| **Sample ID** | **Treatment** | **Time point (h)** |
| --- | --- | --- |
| 10793.Webster.2.F7.160.1181985 | control | 2 |
| 10793.Webster.2.F6.159.1181642 | control | 2 |
| 10793.Webster.2.G6.171.1181096 | control | 24 |
| 10793.Webster.2.G7.172.1182156 | control | 24 |
| 10793.Webster.2.G5.170.1181815 | control | 24 |
| 10793.Webster.2.D8.137.1181526 | WAF 1.6% | 2 |
| 10793.Webster.2.D9.138.1181317 | WAF 1.6% | 2 |
| 10793.Webster.2.E10.151.1181156 | WAF 1.6% | 24 |
| 10793.Webster.2.E8.149.1181293 | WAF 1.6% | 24 |
| 10793.Webster.2.E9.150.1181073 | WAF 1.6% | 24 |
| 10793.Webster.2.F9.162.1181180 | CWAF 1.6% | 2 |
| 10793.Webster.2.F8.161.1182067 | CWAF 1.6% | 2 |
| 10793.Webster.2.F10.163.1182322 | CWAF 1.6% | 2 |
| 10793.Webster.2.G9.174.1182297 | CWAF 1.6% | 24 |
| 10793.Webster.2.G8.173.1181954 | CWAF 1.6% | 24 |
| 10793.Webster.2.E1.142.1181675 | WAF 25% | 2 |
| 10793.Webster.2.D12.141.1181416 | WAF 25% | 2 |
| 10793.Webster.2.D11.140.1182434 | WAF 25% | 2 |
| 10793.Webster.2.E11.152.1181423 | WAF 25% | 24 |
| 10793.Webster.2.F1.154.1182330 | WAF 25% | 24 |
| 10793.Webster.2.E12.153.1181522 | WAF 25% | 24 |
| 10793.Webster.2.F12.165.1182151 | CWAF 25% | 2 |
| 10793.Webster.2.F11.164.1181262 | CWAF 25% | 2 |
| 10793.Webster.2.G1.166.1181397 | CWAF 25% | 2 |
| 10793.Webster.2.G12.177.1181270 | CWAF 25% | 24 |
| 10793.Webster.2.H1.178.1181857 | CWAF 25% | 24 |
| 10793.Webster.2.G11.176.1182132 | CWAF 25% | 24 |
| 10793.Webster.2.G2.167.1181545 | CWAF 50% | 2 |
| 10793.Webster.2.G3.168.1181512 | CWAF 50% | 2 |
| 10793.Webster.2.G4.169.1182328 | CWAF 50% | 2 |
| 10793.Webster.2.H2.179.1182438 | CWAF 50% | 24 |
| 10793.Webster.2.E2.143.1181839 | WAF 100% | 2 |
| 10793.Webster.2.E4.145.1181795 | WAF 100% | 2 |
| 10793.Webster.2.E3.144.1182244 | WAF 100% | 2 |
| 10793.Webster.2.F3.156.1181189 | WAF 100% | 24 |
| 10793.Webster.2.F2.155.1181842 | WAF 100% | 24 |
| 10793.Webster.2.F4.157.1182159 | WAF 100% | 24 |
