# Supplementary material for: The Effects of Crude Oil and Dispersant on the Larval Sponge Holobiont
Source: mSystems. 2019 Dec 10;4(6):e00743-19. doi: 10.1128/mSystems.00743-19 (PMC6906743; doi:10.1128/mSystems.00743-19)
Supplement: TABLE S4 [file mSystems.00743-19-st004.docx]

| **Analyte** | **RL** | **Control** | **WAF** | **CWAF** |
| --- | --- | --- | --- | --- |
|  |  | µg l^-1^ | µg l^-1^ | µg l^-1^ |
| ***PAHs*** |  |  |  |  |
| Napthalene | 0.01 | <0.02 | 79 | 14 |
| Acenaphthene | 0.01 | <0.02 | <0.02 | <0.02 |
| Acenaphthylene | 0.01 | <0.02 | <0.02 | <0.02 |
| Fluorene | 0.01 | <0.02 | 16 | 29 |
| Phenanthrene | 0.01 | <0.02 | 12 | <0.02 |
| Anthracene | 0.01 | <0.02 | <0.02 | <0.02 |
| Fluorene | 0.01 | <0.02 | 0.15 | 29 |
| Pyrene | 0.01 | <0.02 | <0.02 | <0.02 |
| Benzo(b+K)fluoranthene | 0.01 | <0.02 | <0.02 | <0.02 |
| Perylene | 0.01 | <0.02 | 0.04 | 0.21 |
| Benzo(a)pyrene | 0.01 | <0.02 | <0.02 | 0.04 |
| Benzo(e)pyrene | 0.01 | <0.02 | <0.02 | <0.02 |
| Indeno(1,2,3-cd)pyrene | 0.01 | <0.02 | <0.02 | <0.02 |
| Idibenz(a,H)anthracene | 0.01 | <0.02 | <0.02 | <0.02 |
| Benzo(ghi)perylene | 0.01 | <0.02 | <0.02 | 0.07 |
| *100% WAF Total ∑PAHs* |  | *< 0.02* | *107.19* | *72.32* |
|  |  |  |  |  |
| ***TPHs*** |  |  |  |  |
| Hydrocarbons C10-C14 | 10 | <10 | 860 | 32000 |
| Hydrocarbons C15-C28 | 10 | <10 | 3200 | 2000 |
| Hydrocarbons C29-C36 | 10 | <10 | <10 | 200 |
| *TPH* |  | *0* | *4060* | *34200* |
