# Supplementary material for: The Effects of Crude Oil and Dispersant on the Larval Sponge Holobiont
Source: mSystems. 2019 Dec 10;4(6):e00743-19. doi: 10.1128/mSystems.00743-19 (PMC6906743; doi:10.1128/mSystems.00743-19)
Supplement: TABLE S5 [file mSystems.00743-19-st005.docx]

| **Gene name** | **Abbreviation** | **Accession No.** |
| --- | --- | --- |
| YWHAZ | YWHAZ | HS586541 |
| Cyclophilin | Cyp | HS586542 |
| Profilin | Pfn | HS586534 |
| Radia spoke protein | RSP | HS586538 |
| Protein NIMA-interacting 4 | PIN4 | HS586545 |
| YWHAH | YWHAH | HS586539 |
| Actin-related proetin 2/3 complex subunit 4 | ARPC4 | HS097565 |
| Ferritin | Fer | HS097567 |
| Ribosomal protein S9 | RPS9 | HS586547 |
| Elongation factor--Tu | EF-Tu | HS586546 |
| YWHAQ | YWHAQ | HS586540 |
| Polyubiquitin | polyUb | HS586550 |
| β-tubulin | bTub | HS586532 |
| Thioredoxin | Txn | HS586548 |
| B-thymosin | Tb4 | HS586536 |
| α-tubulin | aTub | GW667522 |
| Heat shock protein 90 | Hsp90 | HS097569 |
| Glutathione-S-transferase | GST | HS586549 |
| Actin | act | GW667523 |
| Gelsolin | Gsn | HS586533 |
| Heat shock protein 70 | Hsp70 | HS586543 |
| Villin | Vil1 | HS586537 |
| Ubiquitin-conjugating enzyme | Ubc | HS097570 |
| Apoptosis-linked gene 2 | Alg-2 l | HS586544 |
| Calmodulin | CaM | HS097566 |
| Prolidase | PEPD | HS586535 |
