# Supplementary material for: The Effects of Crude Oil and Dispersant on the Larval Sponge Holobiont
Source: mSystems. 2019 Dec 10;4(6):e00743-19. doi: 10.1128/mSystems.00743-19 (PMC6906743; doi:10.1128/mSystems.00743-19)
Supplement: Supplementary file 6 [file mSystems.00743-19-st003.docx]

sOTU ID	Phylum	Class	Order	Nearest Relative	% Similarity	Accession	Treatment
1655	Chloroflexi	Dehalococcoidia	SAR202 clade	uncultured bacterium clone from *Theonella swinhoei* *	100	KM010296	Control
2005	Proteobacteria	Alphaproteobacteria	Rhodobacterales	*Pseudooceanicola nanhaiensis* strain from sediment	100	MH715064	
5142	Gemmatimonadetes	PAUC43f marine benthic group	unclassified	uncultured bacterium clone from *Theonella* sp.*	100	MF039994	
15475	PAUC43f	unclassified	unclassified	uncultured bacterium clone from *Ectyoplasia ferox* *	99	JN126244	
15484	Proteobacteria	Deltaproteobacteria	Myxococcales	uncultured bacterium clone from *Theonella swinhoei**	99	KM010263	
30675	Acidobacteria	Subgroup 11	unclassified	uncultured bacterium clone from marine sediment	99	KP292482	
30677	Proteobacteria	Gammaproteobacteria	HOC36	uncultured bacterium clone from *Ircinia strobilina* *	98	JX280274	
3859	Proteobacteria	Gammaproteobacteria	KI89A clade	uncultured bacterium clone from *Coscinoderma mathewsi* *	100	JX455224	WAF
3889	Proteobacteria	Gammaproteobacteria	Nitrosococcales	uncultured bacterium clone from *Ectyoplasia ferox* *	100	JN126215	
4173	Poribacteria	unclassified	unclassified	uncultured bacterium clone from *Theonella* sp.*	100	KX519688	
5204	Gemmatimonadetes	BD2-11 terrestrial group	unclassified	uncultured bacterium clone from *Porites lutea*+	100	KC514349	
5272	Chloroflexi	Dehalococcoidia	SAR202 clade	uncultured bacterium clone from *Aplysina cauliformis* *	100	KF286061	
5353	Actinobacteria	Acidimicrobiia	Microtrichales	uncultured bacterium clone from *Porites lutea*+	100	KC514263	
5369	Actinobacteria	Acidimicrobiia	Actinomarinales	uncultured bacterium clone from *Stylophora pistillata*+	100	KC669080	
30622	Poribacteria	unclassified	unclassified	uncultured bacterium clone from *Rhabdastrella globostellata* *	97	KX519669	
215	Proteobacteria	Alphaproteobacteria	unclassified	uncultured bacterium clone from *Geodia barretti* *	100	JQ612330	CWAF
362	Actinobacteria	Acidimicrobiia	Actinomarinales	uncultured bacterium clone from *Porites lutea*+	100	KC514303	
852	Proteobacteria	Gammaproteobacteria	Alteromonadales	uncultured bacterium clone from marine sediment	100	MN463095	
1624	Proteobacteria	Gammaproteobacteria	Enterobacteriales	uncultured bacterium clone from *Theonella* sp.*	100	MF039983	
3808	Chloroflexi	Anaerolineae	Caldilineales	uncultured bacterium clone from *Xestospongia muta* *	100	FJ481375	
6679	Chloroflexi	Dehalococcoidia	SAR202 clade	uncultured bacterium clone from *Ancorina alata* *	100	GU971118	
30628	Chloroflexi	Anaerolineae	SBR1031	uncultured bacterium clone from *Coscinoderma mathewsi* *	99	JX455247	
30644	Proteobacteria	Gammaproteobacteria	KI89A clade	uncultured bacterium clone from *Ircinia* sp.*	100	JX455566	
